# HGTMDA: A Hypergraph Learning Approach with Improved GCN-Transformer for miRNA–Disease Association Prediction

**DOI:** 10.3390/bioengineering11070680

**Published:** 2024-07-04

**Authors:** Daying Lu, Jian Li, Chunhou Zheng, Jinxing Liu, Qi Zhang

**Affiliations:** School of Cyber Science and Engineering, Qufu Normal University, Qufu 273165, China

**Keywords:** miRNA–disease association, GCN-Transformer, random walk with restart, hypergraph learning

## Abstract

Accumulating scientific evidence highlights the pivotal role of miRNA–disease association research in elucidating disease pathogenesis and developing innovative diagnostics. Consequently, accurately identifying disease-associated miRNAs has emerged as a prominent research topic in bioinformatics. Advances in graph neural networks (GNNs) have catalyzed methodological breakthroughs in this field. However, existing methods are often plagued by data noise and struggle to effectively integrate local and global information, which hinders their predictive performance. To address this, we introduce HGTMDA, an innovative hypergraph learning framework that incorporates random walk with restart-based association masking and an enhanced GCN-Transformer model to infer miRNA–disease associations. HGTMDA starts by constructing multiple homogeneous similarity networks. A novel enhancement of our approach is the introduction of a restart-based random walk association masking strategy. By stochastically masking a subset of association data and integrating it with a GCN enhanced by an attention mechanism, this strategy enables better capture of key information, leading to improved information utilization and reduced impact of noisy data. Next, we build an miRNA–disease heterogeneous hypergraph and adopt an improved GCN-Transformer encoder to effectively solve the effective extraction of local and global information. Lastly, we utilize a combined Dice cross-entropy (DCE) loss function to guide the model training and optimize its performance. To evaluate the performance of HGTMDA, comprehensive comparisons were conducted with state-of-the-art methods. Additionally, in-depth case studies on lung cancer and colorectal cancer were performed. The results demonstrate HGTMDA’s outstanding performance across various metrics and its exceptional effectiveness in real-world application scenarios, highlighting the advantages and value of this method.

## 1. Introduction

MicroRNAs (miRNAs) are short, non-coding RNA molecules, usually 20–24 nucleotides long, that originate from longer primary transcripts (pri-miRNAs) via a series of processing steps. miRNAs play a pivotal role in cellular regulation, regulating multiple biological processes, including cell proliferation, differentiation, development, metabolism, and immunity [1]. The essential functions of miRNAs make them closely associated with the onset and progression of numerous diseases [2]. For instance, in cardiovascular diseases, miRNAs are considered potential therapeutic targets and diagnostic biomarkers [3]. Extensive research has revealed the critical role of miRNAs in cancer development, highlighting their role as potential oncogenes or tumor suppressors during the development and progression of tumors [4]. Lung and colorectal cancers are major contributors to cancer mortality worldwide. Although diagnostic and therapeutic strategies have improved, the outlook for patients diagnosed at advanced stages remains bleak. Given the pivotal role of miRNAs in cancer initiation and progression, there is a pressing need to develop innovative predictive and diagnostic approaches for early detection and tailored treatment. Precise identification of cancer-linked miRNAs lays the foundation for further biological investigations and clinical applications, offering hope for enhancing patient care and outcomes. Considering the crucial role of miRNAs in diseases, thoroughly investigating miRNA–disease associations is of paramount importance for advancing human health [5]. Exploring miRNA–disease relationships not only helps elucidate disease mechanisms at the molecular level but also offers insights for developing novel diagnostic and therapeutic approaches. As research continues to deepen, experimental techniques can directly detect miRNA–disease associations, yielding reliable results that are widely recognized by medical researchers. However, experimental methods suffer from drawbacks such as high costs and time-consuming processes. Considering the vast number of miRNA–disease correlations, relying solely on experimental means to validate each association is impractical. In contrast, computational approaches can utilize established benchmark databases to identify potential miRNA–disease associations more cost-effectively and efficiently. These computational approaches complement experimental methods, offering valuable research leads for biologists and medical researchers. Recently, deep learning methods have emerged as promising approaches for miRNA–disease association estimation (MDA). Existing MDA prediction methods can be generally classified into two categories: similarity-based and machine learning-based approaches.

Similarity-based methods rely on a fundamental assumption: functionally similar miRNAs are likely to be associated with diseases that share similar clinical characteristics. For instance, Liang et al. [6] employed the k-nearest neighbors algorithm to construct graph structures representing miRNA and disease similarities, effectively extracting key information from these similarities. Chen et al. [7] developed an innovative algorithm that employs a random walk with restart mechanism to identify the crucial roles of miRNAs in disease onset and progression. The distinguishing feature of this method is its utilization of the entire network, rather than just local regions, to measure similarity. Xuan et al. [8] developed a weighted k-nearest neighbors algorithm that assigns higher weights to members of the same miRNA family when predicting miRNA–disease crosstalk. Jiang et al. [9] employed kernel fusion techniques to integrate various types of miRNA and disease similarity matrices. However, models that heavily rely on similarity scores may overemphasize these scores when predicting miRNA–disease associations, potentially leading to certain limitations in the prediction process.

Alternatively, with the increasing prevalence of artificial intelligence, numerous machine learning-based techniques have gradually been introduced into the study of interactions between miRNAs and diseases [10]. Fu and his research team utilized deep autoencoder techniques to extract core information from information-rich networks and employed neural network classification units to predict associations [11]. In another study, Chen et al. [12] obtained feature properties from miRNA and disease similarities and introduced the random forest (RF) algorithm for constructing a predictive model, thereby inferring potential connections between miRNAs and diseases. Peng et al. [13] proposed a convolutional neural network-based prediction model that utilizes autoencoders to extract shared features between miRNAs and diseases, enabling the prediction of their interactions. Furthermore, Li et al. [14] introduced MCMDA, a model that utilizes singular value thresholding techniques for miRNA–disease association prediction and optimizes the miRNA–disease adjacency matrix using matrix completion methods to generate the final association matrix. Overall, miRNA–disease association prediction models that leverage machine learning techniques have demonstrated high efficiency and significantly reduced computational resource requirements. It is important to acknowledge that the model’s feature extraction performance is critical in determining the accuracy of the final predictions.

Predicting miRNA–disease association (MDA) can be formulated as a link prediction problem in a bimodal network, which is well suited for GNNs [15]. This need has catalyzed the swift advancement of numerous GNN-based MDA prediction models. Exemplifying this, Li et al. [16] proposed a graph-based autoencoder framework to learn latent feature representations of miRNAs and diseases from the miRNA–disease association bipartite graph. In a separate study, Li’s group [17] built a heterogeneous graph incorporating miRNA similarity, disease similarity, and known association information, employing graph convolutional autoencoder techniques to reconstruct and uncover these associations. Despite the development of various MDA prediction methods, there remain some challenges to be addressed. Firstly, while existing GNN models can integrate more comprehensive node information to enhance the performance of predicting miRNA–disease associations, these models may overly rely on existing connections and features of neighboring nodes in the network, leading to prediction bias. Secondly, existing models still have limitations in effectively integrating local structural information and global dependencies. Lastly, current models do not adequately consider the handling of noisy data, which may limit their predictive performance.

Inspired by the work of Ning et al. [18], this study proposes an innovative miRNA–disease association prediction method called HGTMDA. Our approach introduces several key improvements compared to previous methods. First, we employ a restart random walk-based association masking strategy, coupled with attention-enhanced GCNs, to effectively reduce data noise and strengthen information extraction. Second, we utilize a GCN-Transformer encoder for the heterogeneous hypergraph, enabling better capture of local structural features and global dependencies. Finally, we adopt the DCE loss, combining Dice and cross-entropy losses, to optimize model performance more comprehensively. These innovations contribute to the enhanced accuracy and robustness of our method in predicting miRNA–disease associations. The key contributions of this approach are as follows:

- An enhanced GCN-Transformer framework is adopted to effectively integrate local structural information and global dependencies, capturing complex interactions and hierarchical relationships between nodes through multi-scale aggregation and update operations within each encoder layer.
- A novel restart-based random walk association masking strategy is introduced and integrated with an attention-enhanced GCN, effectively reducing data noise while strengthening information extraction.
- Introducing the DCE loss function, which addresses class imbalance issues and probability distribution differences, improving the model’s generalization ability and convergence speed, thereby optimizing model performance more comprehensively.
- Conducting experimental validations on multiple benchmark datasets. The analysis reveals that HGTMDA outperforms existing methods, demonstrating its efficacy and superiority in predicting miRNA–disease associations.

## 2. Materials and Methods

### 2.1. Datasets

To thoroughly investigate the associations between miRNAs and diseases, we obtained known MDA association data from the Human MicroRNA Disease Database (HMDD v3.2) [19]. This database has collected a large number of experimentally verified miRNA–disease interconnections from the published literature through manual curation and text mining techniques. After careful screening [20], we ultimately identified 853 miRNAs and 591 diseases, with 12,446 known associations between them [21]. In our experiments, we treat the 12,446 known miRNA–disease associations from the HMDD v3.2 database as positive samples. To tackle the sparsity of positive samples in the association graph, we balance positive and negative samples by randomly selecting an equal number of data points from unknown and known non-associated samples as negative samples. This approach creates a balanced dataset with equal positive and negative samples. We divided the HMDD v3.2 dataset into a training set and an independent test set using an 8:2 ratio. In the data preprocessing stage, known MDA associations were assigned a positive label (denoted as 1), while the remaining unknown associations were designated as negative samples (denoted as 0). The hypothesis that “functionally similar miRNAs are likely to be associated with phenotypically similar diseases” underscores the importance of incorporating similarity information surrounded by diseases and surrounded by miRNAs when predicting novel miRNA–disease associations. By deeply analyzing these two types of similarity relationships, we can more accurately infer which specific diseases a particular miRNA is likely to be associated with, providing important clues for elucidating the role of miRNAs in disease occurrence and development. Consequently, comprehensive integration of disease and miRNA similarity information is crucial in miRNA–disease synergy prediction studies, as it enhances the model’s predictive performance and interpretability.

**Figure 1:**
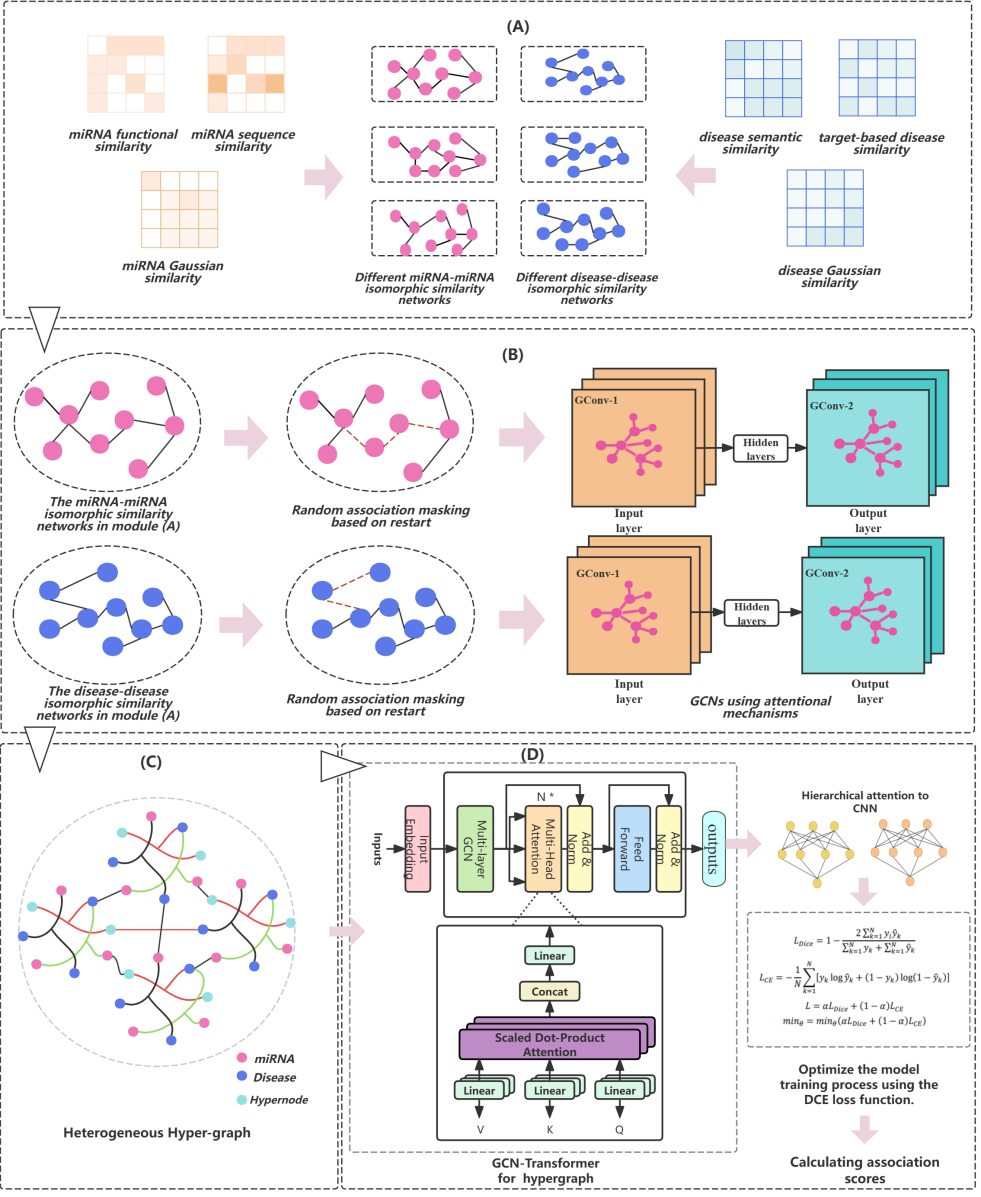
Overall architecture of HGTMDA. (**A**) Construction of miRNA and disease similarity networks. (**B**) Association masking and feature extraction. (**C**) Association masking and feature extraction. (**D**) Information aggregation and association prediction.

Inspired by previous research, we designed an innovative approach for miRNA–disease association prediction named HGTMDA. As shown in Figure 1, HGTMDA comprises several key modules:

(A) An isomorphic similarity network is generated by leveraging the collected miRNA and disease similarity data.
(B) For both miRNA and disease isomorphic networks, association masking is performed based on random walks with restart, randomly masking some network connections. Subsequently, feature extraction on the masked networks is carried out using graph convolutional networks (GCNs) with an introduced attention mechanism.
(C) By introducing the concept of supernodes, we construct an miRNA–disease association (MDA) heterogeneous hypergraph. Then, GCN-Transformer is utilized to aggregate and integrate information within the heterogeneous hypergraph.
(D) The graph neural networks’ aggregated output is combined, and the model’s loss is computed using the DCE loss function, which guides the model’s optimization and parameter learning process.

### 2.2. Constructing Homogeneous Similarity Networks

To investigate similarity information that can aid in association prediction, we initially process various aspects of miRNA and disease similarity. Within the scope of this analysis, multiple similarity metrics are employed to fabricate miRNA–miRNA and disease–disease interaction networks. For miRNAs, we consider functional similarity, sequence similarity, and Gaussian interaction profile kernel similarity, each of which is used to generate an adjacency matrix for a distinct miRNA–miRNA network. Likewise, to compute the similarity between diseases, we employ three measurement approaches: semantic similarity index, goal-directed similarity, and Gaussian interaction profile kernel matching degree. These three similarity indicators are utilized to construct three distinct disease–disease networks, and their respective adjacency matrices are utilized in the subsequent analysis. In summary, the adjacency matrices for miRNAs and diseases can be, respectively, divided into

(1) $$A_{m}=\left\{A_{mf},\;A_{ms},\;A_{mg}\;\right\}$$

(2) $$A_{d}=\left\{A_{ds},\;A_{dt},\;A_{dg}\;\right\}$$

The sets $\left(A_{mf,}\;A_{ms,}\;A_{mg}\right)\;\in R^{k_{m}\times k_{m}}$ and $\left(A_{ds},\;A_{dt},\;A_{dg}\right)\;\in R^{k_{d}\times k_{d}}$, where $A_{mf}$, $A_{ms}$, and $A_{mg}$ represent the adjacency matrices of different miRNA–miRNA interaction networks, and $A_{ds}$, $A_{dt}$, and $A_{dg}$ represent the adjacency matrices of different disease–disease interaction networks; where $k_{m}$ and $k_{d}$ denote the number of miRNAs and diseases, respectively. For detailed information on the various similarity adjacency matrices, please refer to the Supplementary Materials.

### 2.3. Random Association Masking and Information Extraction

This study presents a novel approach that employs a restart-enabled random walk strategy on the homogeneous similarity networks of miRNAs and diseases to generate masks for obscuring specific associations. This strategy helps the model avoid excessive dependence on specific associations, enhancing its robustness. Subsequently, the GCN can more effectively extract critical information from the miRNA and disease nodes, thereby improving the overall performance and effectiveness of the method. The primary steps include initially determining whether to select a node from the homogeneous similarity network as the starting point based on a Bernoulli distribution. We chose the Bernoulli distribution for several key reasons. First, it perfectly suits our research scenario of determining whether to select a node as the starting point for random walks. Second, the Bernoulli distribution’s single parameter p, representing the “success” probability, allows flexible control over the probability of selecting a node as the starting point. This adaptability is crucial for accommodating different datasets and task requirements. Furthermore, the Bernoulli distribution is computationally more efficient compared to alternatives like Gaussian or Poisson distributions. This efficiency significantly reduces computational overhead and enhances the algorithm’s scalability when dealing with large-scale graph data.

(3) $$\begin{array}{c} S∼Bernoulli(p) \end{array}$$

In this context, *S* denotes the collection of nodes sampled from the graph based on a Bernoulli distribution, where *p* indicates the sampling rate, which is bounded by 0 and 1. We then employ a restart-enabled random walk strategy to extract associations within the network. The transition probability matrix for the random walk is defined as follows:

(4) $$P=\left(1-c\right)D^{-1}A+cI$$

(5) $$path\left(v_{i}\right)=\left[\begin{array}{c} v_{i},v_{i_{1}},v_{i_{2}},\ldots ,v_{i_{k}} \end{array}\right]$$

In the equation, *c* represents the restart probability; *D* represents the number of connections for each node in the graph, where diagonal elements indicate the degree of the nodes and off-diagonal elements are zero; *I* denotes the identity matrix; $path(v_{i})$ represents the random walk path originating from node $\left(v_{i}\right)$ in the homogeneous similarity network; and *k* is the path length.

Then, for each extracted path $path(v_{i})$, we generate a corresponding binary mask vector $m\left(v_{i}\right)\in {\left\{0,1\right\}}^{n}$. Each element $m_{j}\left(v_{i}\right)$ of the mask vector indicates whether the node $v_{j}$ is included in the path, that is,

(6) $$\begin{array}{c} m_{j}\left(v_{i}\right)=\left\{\begin{array}{cc} 1, & if\;v_{j}\in \mathrm{path}\left(v_{i}\right)\\ 0, & \mathrm{otherwise} \end{array}\right. \end{array}$$

Subsequently, we apply the generated path masks to the original adjacency matrix, thereby obtaining the masked adjacency matrix:

(7) $$\begin{array}{c} A^{\left(mask\right)}=A⊙\left\{\cup_{v_{i\in S}}m\left(v_{i}\right)m{\left(v_{i}\right)}^{T}\right\} \end{array}$$

Concurrently, a graph convolutional network (GCN) integrated with an attention mechanism is employed to extract information from the network following path masking. GCN, a deep learning model specifically developed for processing graph-structured data, is utilized in this study. The GCN learns node representations by aggregating neighborhood information, while the attention mechanism enables the model to assign varying weights based on the relevance of the input, thereby more effectively capturing key information. By integrating the neighborhood aggregation of GCN and the weighted distribution of attention, we learn the network’s critical structure and node importance.

### 2.4. Construction of Heterogeneous Hypergraphs

To boost the estimation of potential miRNA–disease associations, we develop an miRNA–disease hypergraph using supernodes after obtaining miRNA and disease embeddings that aggregate multi-source similarity information [18]. In this hypergraph model, the virtual node is coupled with all miRNA and disease nodes, encompassing both known and unknown miRNA–disease associations. The introduction of supernodes allows for a deeper exploration of the possible links between miRNAs and diseases. Hypernode embeddings are obtained through a self-learning mechanism, allowing them to adaptively extract the most informative features from miRNA and disease embeddings. Each hypernode’s $Q_{k}$ embedding vector is randomly initialized as (1 × E)-dimensional, and then, updated via a neural network model based on the embeddings of miRNA nodes, disease nodes, and their complex relationships. This self-learning process enables hypernodes to adaptively encode relevant information from the miRNA–disease network. By employing this network-based approach, hypernodes can establish high-quality connections between miRNAs and diseases, leading to more accurate association score predictions.

When constructing the hypergraph, we employ cosine similarity to compute the adjacency matrix. Cosine similarity effectively measures the degree of similarity between miRNA nodes and disease nodes, providing appropriate weight information for the construction of the hypergraph. The formula is as follows:

(8) $$C_{M_{i,}\;Q_{k}}=M_{i}\cdot Q_{k}\;/\;(∥M_{i}∥∥Q_{k}∥)$$

(9) $$C_{D_{j,}\;Q_{k}}=M_{i}\cdot Q_{k}\;/\;(∥D_{j}∥∥Q_{k}∥)$$

where $M_{i}$ and $D_{j}$ represent the node embeddings for miRNA and disease, respectively, and $Q_{k}$ is the supernode embedding learned through the network-based self-learning mechanism. ||·|| denotes the L2 norm. $C_{M_{i,}\;Q_{k}}$ and $C_{D_{j,}\;Q_{k}}$ indicate the proximity of $M_{i}$ and $D_{j}$ to the supernode $Q_{k}$ within a common feature space, reflecting their potential association likelihood.

Inspired by previous work [22], we adopt a GCN-Transformer encoder tailored for our hypergraph structure. Unlike traditional transformers, the GCN-Transformer effectively handles graph-structured data by organically combining GCNs and transformers, capturing both local structural features and modeling global dependencies. In this study, the encoder employs multiple stacked GCN-Transformer encoder layers to form the complete encoder. Each encoder layer consists of separate GCN layers and the self-attention mechanism of transformers. The computation formula for the GCN layer is as follows:

(10) $${\hat{A}}_{l}=D^{-\frac{1}{2}}\cdot A_{l}\cdot D^{-\frac{1}{2}}$$

(11) $$H^{\left(r+1\right)}=\sigma \left({\hat{A}}_{l}\cdot H^{\left(r\right)}\cdot W^{\left(r\right)}\right)$$

${\hat{A}}_{l}$ represents the normalized association matrix, *D* is the diagonal matrix characterizing the node connectivity, $\;H^{\left(r\right)}$ denotes the node feature matrix at layer *(r)*, and $W^{\left(r\right)}$ signifies the weight matrix at layer *r*. The ReLU activation function is denoted by σ.

To focus more on neighboring nodes and enhance the attention on local information, we introduce a node distance matrix as a bias term in the self-attention mechanism. The node distance matrix $D_{\varphi (G)}$ captures the spatial proximity among graph nodes, quantified by the minimum path length connecting any node pair. By incorporating it as a bias term in the self-attention computation, the model can better capture the spatial dependencies between nodes, rather than solely relying on the similarity of node features. Since neighboring nodes tend to have stronger associations, the shortest path matrix enables the self-attention mechanism to pay more attention to these nodes when computing attention weights. The computation formula for the transformer’s self-attention mechanism is as follows:

(12) $$Q^{\left(r\right)}=H^{\left(r\right)}W_{Q}^{\left(r\right)},K^{\left(r\right)}=H^{\left(r\right)}W_{K}^{\left(r\right)},V^{\left(r\right)}=H^{\left(r\right)}W_{V}^{\left(r\right)}$$

(13) $$Attention\left(Q^{\left(r\right)},K^{\left(r\right)},V^{\left(r\right)}\right)=softmax\left(\frac{Q^{\left(r\right)}{\left(K^{\left(r\right)}\right)}^{T}}{\sqrt{d_{k}}}+D_{\varphi (G)}\right)V^{\left(r\right)}$$

(14) $${\tilde{H}}^{\left(r\right)}=Attention\left(Q^{\left(r\right)},K^{\left(r\right)},V^{\left(r\right)}\right)$$

In the equations, $Q^{\left(r\right)}$, $K^{\left(r\right)}$, and $V^{\left(r\right)}$ are the query, key, and value matrices, respectively, while $W_{Q}^{\left(r\right)}$, $W_{K}^{\left(r\right)}$, and $W_{V}^{\left(r\right)}$ represent the corresponding weight matrix. $d_{k}$ denotes the dimension of the key vector and $D_{\varphi (G)}$ is the bias matrix based on the shortest path.

Additionally, we introduce residual connections and layer normalization (layer normalization) to the encoder, and apply the multi-head attention mechanism to the GCN-Transformer encoder layers. Residual connections allow input features to be directly propagated across different layers of the network, preventing features from being completely altered or lost. Meanwhile, layer normalization ensures that the output distribution of residual connections remains stable, thereby preventing gradient vanishing when information is propagated through deep layers of the network.

(15) $$H^{\left(r+1\right)}=LN\left(H^{\left(r\right)}+{\tilde{H}}^{\left(r\right)}\right)$$

(16) $$MultiHead\left(H^{\left(r\right)}\right)=Concat\left(head_{1},\ldots ,head_{h}\right)W_{O}^{\left(r\right)}$$

(17) $$head_{i}=Attention\left(H^{\left(r\right)}W_{Qi}^{\left(r\right)},H^{\left(r\right)}W_{Ki}^{\left(r\right)},H^{\left(r\right)}W_{Vi}^{\left(r\right)}\right)$$

In the equations, *LN* denotes the layer normalization operation, $head_{i}$ represents the *i*-th attention head, $W_{Qi}^{\left(r\right)}$, $W_{Ki}^{\left(r\right)}$, and $W_{Vi}^{\left(r\right)}$ represent the weight matrix corresponding to the *i*-th attention head, and $W_{O}^{\left(r\right)}$ is the weight matrix for the output linear transformation.

Finally, we perform iterative updates on the miRNA and disease nodes.

(18) $$Z_{m}=GCTEncoder\left(X_{m}\right)$$

(19) $$Z_{d}=GCTEncoder\left(X_{d}\right)$$

In the equations, $X_{m}$ and $X_{d}$ represent the initial feature matrices for miRNA nodes and disease nodes, respectively, while $Z_{m}$ and $Z_{d}$ denote the learned node representations for miRNAs and diseases.

### 2.5. Calculating the Loss

After obtaining the miRNA and disease node representations from the heterogeneous hypergraph, an attention mechanism is used to integrate these representations with varying weights. The Hadamard product of the miRNA vector representations ${\hat{m}}_{i}$ and the disease embeddings ${\hat{d}}_{i}$ is then calculated and input into a single-layer feedforward neural network (FNN) activated by a Sigmoid function to obtain the probability of association between miRNAs and diseases, as expressed by the following formula:

(20) $$\begin{array}{c} {\hat{y}}_{t}=FNN\left[cnn\left(a({\hat{m}}_{i})\right)⊙cnn\left(a({\hat{d}}_{i})\right)\right] \end{array}$$

where (*a*) represents the attention mechanism, and cnn(·) denotes a 1D convolutional neural network (CNN).

During the training process, a combined loss function that integrates the Dice loss and cross-entropy loss functions is adopted to optimize our model. The Dice loss function is better suited for handling class imbalance issues, offering greater penalties for prediction errors on minority class samples, thereby enhancing the model’s generalization capabilities. However, it has a higher computational complexity, which may lead to longer training times. The cross-entropy loss function, on the other hand, more effectively measures the discrepancy between the predicted and true probability distributions, facilitating faster model convergence but with weaker capabilities in addressing class imbalance issues, potentially leading the model to favor majority classes. By combining these two loss functions, we take into account class imbalance while also considering probability distribution discrepancies, thereby more comprehensively optimizing model performance, enhancing both the model’s generalization ability and convergence speed.

The Dice loss function is expressed as

(21) $$\begin{array}{c} L_{Dice}=1-\frac{2\sum_{t=1}^{N}y_{t}{\hat{y}}_{t}}{\sum_{t=1}^{N}y_{t}+\sum_{t=1}^{N}{\hat{y}}_{t}} \end{array}$$

where *(N)* is the total number of samples. The Dice loss function values range from [0, 1], with smaller values indicating more accurate predictions.

The cross-entropy loss function is defined as

(22) $$\begin{array}{c} L_{CE}=-\frac{1}{N}\sum_{t=1}^{N}\left[y_{t}\log{\hat{y}}_{t}+\left(1-y_{t}\right)\log\left(1-{\hat{y}}_{t}\right)\right] \end{array}$$

The ultimate objective function is formulated as follows:

(23) $$L=\alpha L_{Dice}+\left(1-\alpha \right)L_{CE}$$

(24) $$min_{\theta }=min_{\theta }\left(\alpha L_{Dice}+\left(1-\alpha \right)L_{CE}\right)$$

In the equation, α∈[0,1] is a weight coefficient that balances the two loss functions, while θ represents the model’s trainable parameters, which are continuously updated through gradient descent.

## 3. Results and Discussion

### 3.1. Comparative Analysis with State-of-the-Art Methods

In this section, we compare HGTMDA with five other methods, namely, NIMCGCN [23], AGAEMD [24], MINIMDA [25], MAGCN [26], and AMHMDA [18], on the HMDD V3.2 dataset. For a fair comparison, the original settings and parameters of the other methods were maintained. Additionally, we incorporate the functionality to dynamically obtain specific similarities as needed, ensuring consistency and reliability in the comparison. By following this approach, we compare the performance of different methods under the same conditions.

- NIMCGCN [23]: This approach utilizes graph convolutional networks (GCNs) to acquire node embeddings from similarity networks. The obtained node representations are then input into a matrix completion model (NIMC). By optimizing the objective function, a complete association matrix is generated.
- AGAEMD [24]: In the study of constructing miRNA–disease association networks, this approach integrates information by applying an encoder that focuses on node importance, thereby reconstructing and optimizing the interaction network between miRNAs and diseases.
- MINIMDA [25]: This technique comprehensively fuses the high-order adjacency information from multiple data type networks by creating network structures. Through this process, it learns the intrinsic representations between miRNAs and diseases.
- MAGCN [26]: By leveraging the interactions between lncRNAs and miRNAs, this method employs a hybrid approach that combines an attention mechanism-infused graph convolutional network and convolutional neural network to predict undiscovered miRNA–disease interplay.
- AMHMDA [18]: This approach creates an miRNA–disease heterogeneous hypergraph through a virtual hypernode and utilizes graph convolutional networks (GCNs) to aggregate information, thereby inferring the miRNA–disease relationships.

**Figure 2:**
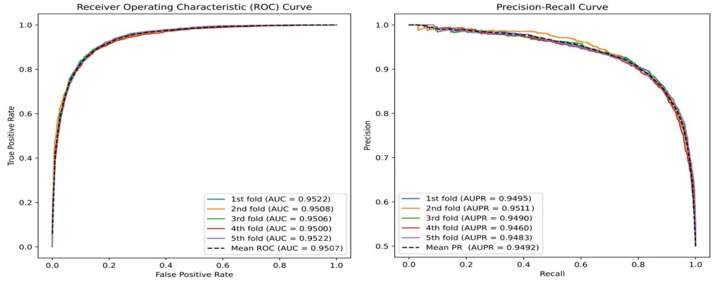
The 5-CV ROC and PR curves.

**Figure 3:**
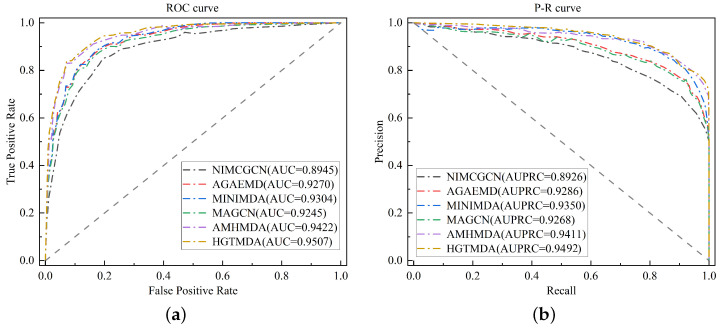
(**a**) Comparative results of AUC for various methods. (**b**) Comparative results of AUPRC for various methods.

Through multiple experiments, we obtained the validation results of HGTMDA. As presented in Table 1, the results demonstrate that HGTMDA achieves an average AUC of 0.9507 and an average AUPR of 0.9492, as depicted in Figure 2, surpassing other state-of-the-art models. To highlight the superior performance of our model, we compared HGTMDA with the remaining models, and these discoveries are presented in the form of comparative graphs, as depicted in Figure 3. The graphs clearly demonstrate the excellence of our method, which is inextricably linked to the innovative approaches we employed, namely, the restart-based random walk association masking and the combined loss function. The comparative results underscore the advantages of our proposed methodology.

### 3.2. Ablation Experiments

To further demonstrate the efficacy of HGTMDA, we modified the model and obtained four variants: HGT-A, HGT-B, HGT-C, and HGT-D. Specifically, for HGT-A, we removed the random walk association masking with restart module to test the impact of the association masking module on model performance and robustness. HGT-B represents our model using the association masking module without restarts. HGT-C represents our model using traditional GCN instead of the GCN-Transformer encoder. HGT-D was developed by substituting the DCE loss with the standard BCE loss function, which is commonly used in MDA association prediction. This modification allowed us to evaluate the impact of the combined loss function on addressing class imbalance and probability distribution variations. We then compared these four variants with HGTMDA. As illustrated in Table 2, the experimental results highlight the importance of these novel methods in enhancing the model’s ability to identify complex relationships and correlations present in the data. By leveraging the association masking module, HGTMDA effectively mitigates the impact of noise and improves model robustness, enabling it to handle complex real-world scenarios. The GCN-Transformer module effectively integrates local and global information. Furthermore, the integration of the DCE loss function allows the model to effectively address class imbalance and probability distribution discrepancies, leading to more comprehensive and accurate predictions. The results underscore the significance of meticulously developing and incorporating sophisticated techniques to enhance the effectiveness of models that predict miRNA–disease interdependencies.

### 3.3. Case Study

Globally, lung cancer is the primary cause of cancer-related mortality [27], with a significantly low survival rate within a few years after diagnosis. Current research indicates that alterations in the expression of specific miRNAs are strongly closely related to the formation of lung cancer [28]. For example, miR-155 is highly expressed in lung cancer tissues [29] and may promote lung cancer development by regulating multiple tumor suppressor genes. Colorectal cancer ranks among the most prevalent and highly lethal malignancies globally [30], and its occurrence is associated with various factors, including genomic mutations, epigenetic changes, and abnormal activation of related signaling pathways [31]. Research indicates that miR-21 [32] is upregulated in colorectal cancer tissues, potentially exerting oncogenic effects and correlating with poor prognosis.

**Figure 4:**
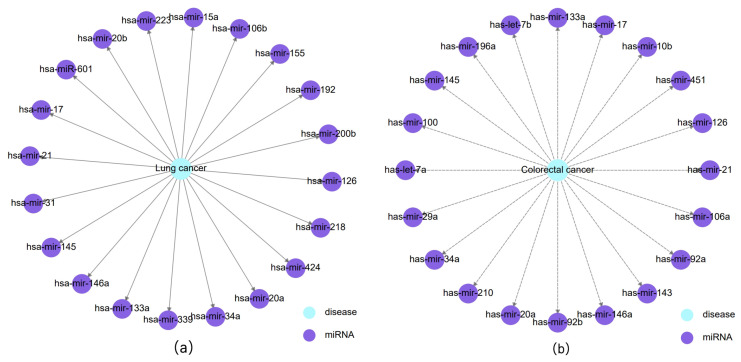
(**a**) Top 20 miRNAs associated with lung cancer predicted by the model. (**b**) Top 20 miRNAs associated with colorectal cancer predicted by the model.

To validate the performance of HGTMDA in practical MDA association prediction, we applied our model to two types of diseases: lung tumors and colorectal tumors. Positive training samples were derived from confirmed miRNA–disease connections in the HMDD V3.2 database, while negative samples consisted of an equal number of unknown interdependencies, excluding those related to the specific diseases in the case studies. The model’s predictions for the top 20 miRNAs associated with the two diseases, ranked by their interdependency scores, are depicted in Table 3 and Figure 4. We used the dbDEMC3.0 [33] database to validate the predicted miRNAs for both diseases, and all of them were confirmed in the database, further demonstrating the reliability and excellent performance of our model.

## 4. Parameter Discussion

### 4.1. Evaluation Metrics

In the experiments, we divided the known MDA associations from the HDMM V3.2 database into training and testing sets. We used five-fold cross-validation to assess HGTMDA’s generalization ability and employed multiple evaluation metrics for a comprehensive performance assessment. The hit rate–false alarm rate curve (ROC) and the integrated area of the curve (AUC) served to critique the model’s overall classification ability at different thresholds. The precision–recall (PR) curve and the area under the curve (AUPRC) were used to measure the balance between precision and retrieval rate across various thresholds. Accuracy was used to reflect the model’s prediction correctness on the entire dataset, while the F1 score provided a balanced performance metric considering both precision and recall. Recall and precision were used to assess the model’s ability to identify positive samples and the accuracy of predicting positive samples, respectively.

### 4.2. Parametric Analysis

In deep learning methods, appropriate hyperparameter configuration is crucial for the model to capture the complex patterns of miRNA–disease associations and perform well in predicting unknown associations. To achieve the best model performance and generalization ability, we conducted a series of experiments to explore the impact of different hyperparameter combinations. After experimental exploration, we finally determined the following hyperparameter settings: for the GCN-Transformer, we set the number of multi-head attention heads to four and the number of GCN layers to two. The maximum path length was set to eight, and the number of neurons in the hidden layer of the feedforward neural network was set to 2048. The strategy mask ratio was set to 0.3, the restart probability was set to 0.6. Through a series of experimental validations, we determined that setting the number of hypernodes to 64 yielded the best performance. Additionally, we introduced dropout regularization with a dropout rate of 0.5 to alleviate overfitting and enhance the model’s generalization capability.

#### 4.2.1. The Impact of the Restart Probability (c)

**Figure 5:**
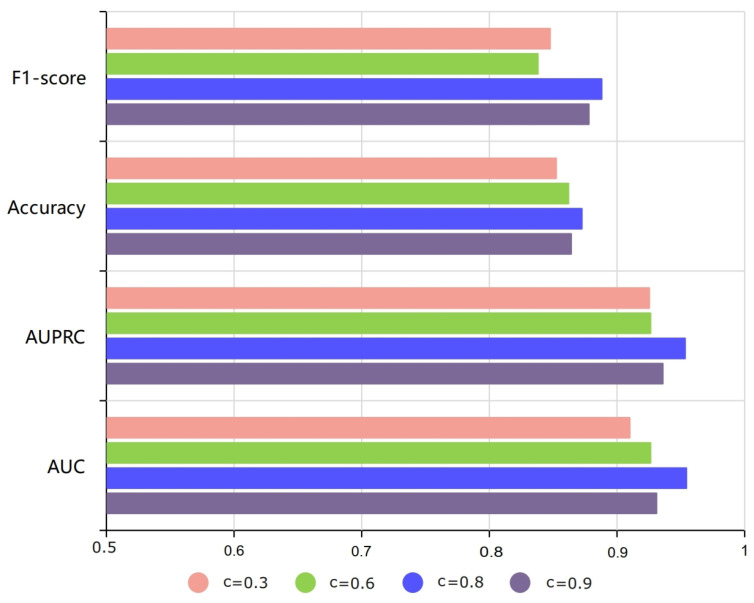
Experimental results for various restart probabilities.

The restart probability significantly influences model performance when employing the random walk with restart-based association masking strategy. In our experiments, we kept the masking ratio and other settings unchanged. Figure 5 shows the model’s performance under different restart probabilities 0.3, 0.6, 0.8, 0.9. The model attains optimal performance when c = 0.6.

Our analysis suggests that a higher restart probability helps suppress noise and enhances the model’s ability to capture local information. This is because the random walk returns to the starting point more frequently, which assists the model in better understanding and utilizing the neighborhood environment of nodes. However, an excessively high restart probability may cause the model to overly rely on local information, potentially leading to the oversight of important global contextual clues. On the other hand, a lower restart probability can reveal more global structures and deeper associations, but it simultaneously increases the risk of noisy data influencing the model’s judgment.

#### 4.2.2. The Impact of the Strategy Mask Ratio (*p*)

**Figure 6:**
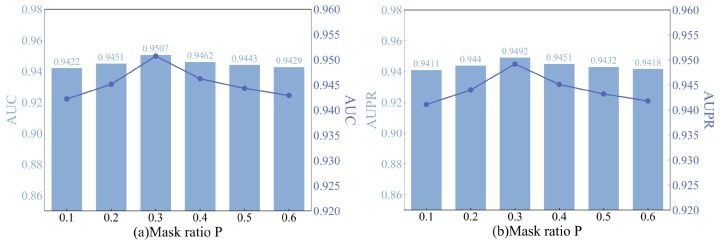
(**a**) AUC comparison graph for different masking ratios. (**b**) AUPR comparison graph for different masking ratios.

The masking ratio *p* represents the probability of sampling starting nodes from the graph. Our designed association masking with restart randomly masks the associations in the single-type node networks of miRNAs and diseases. In the experiments, multiple rounds of experiments were conducted for each *p* value, while keeping the remaining settings unchanged. The experimental findings are presented in Figure 6. For *p* values in the [0.1, 0.3] range, increasing the masking ratio leads to continuous improvement in model performance. Particularly, when *p* = 0.3, the AUC metric reaches its optimum. However, once *p* exceeds 0.4, the model’s performance begins to decline with changes in the masking ratio. This pattern suggests that lower masking ratios effectively reduce the adverse effects of noisy data during self-supervised training. While higher masking ratios may suppress noise, they can also result in the loss of crucial information. Therefore, when setting the masking ratio, it is advisable to avoid excessively high or low *p* values to strike a balance between noise reduction and information preservation.

#### 4.2.3. The Impact of the DCE Loss Parameter (a)

**Figure 7:**
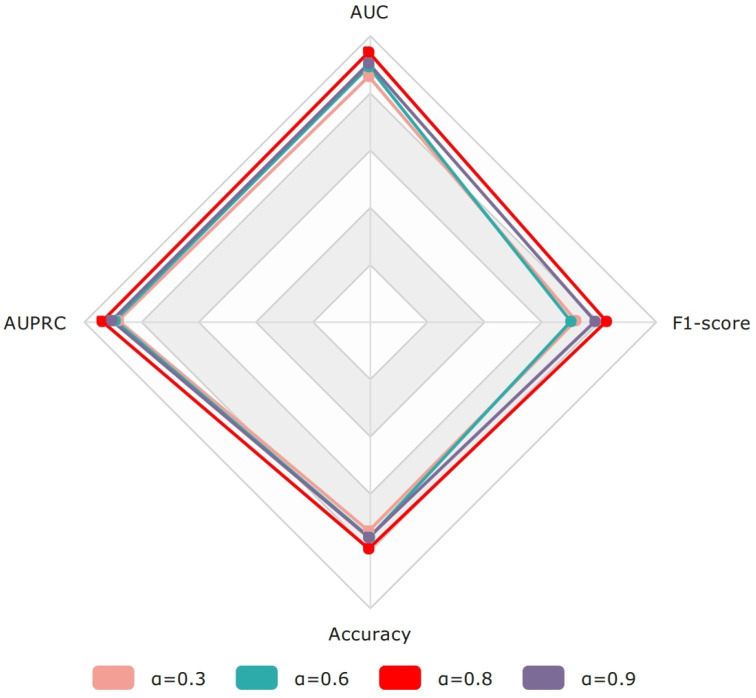
The impact of different weighting coefficients (α) on model performance.

In this study, we employ a combination of two loss functions to calculate the model’s loss. The weight coefficient α plays a crucial role in balancing the two loss functions. An appropriate α value enables the loss function to take into account both class imbalance and probability distribution differences, thereby improving the model’s performance. Therefore, we further investigate the impact of α on the prediction results. Figure 7 illustrates the influence of different α values (0.3, 0.6, 0.8, 0.9) on the model. Our findings suggest that setting the weight coefficient α to 0.8 strikes an optimal balance between the two loss functions, effectively addressing the challenges posed by class imbalance and probability distribution discrepancies. Carefully tuning this hyperparameter ensures that the model can learn from the data more comprehensively and robustly, ultimately improving its predictive capabilities and generalization ability.

#### 4.2.4. The Impact of the Number of Attention Heads and GCN Layers

**Figure 8:**
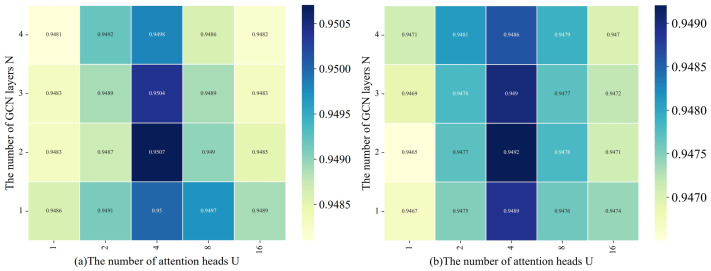
(**a**) Variation in AUC values with U and N. (**b**) Variation in AUPR values with U and N.

To investigate the impact of the number of attention heads and GCN layers on model performance, we conducted a series of comparative experiments. An appropriate number of attention heads aids the model in capturing important features, but excessive heads may introduce irrelevant information and increase complexity. Meanwhile, increasing the number of GCN layers enables the integration of more comprehensive node information, but too many layers can lead to higher computational complexity and potentially cause over-smoothing or overfitting. We evaluated the model’s performance by varying the number of attention heads U and GCN layers N, with the experimental outcomes depicted in Figure 8. When U = 4 and N = 2, the model performance was relatively optimal, as this configuration struck a balance between capturing features and controlling complexity.

## 5. Conclusions

Accurately predicting the interconnections between miRNAs and diseases is crucial for elucidating the molecular mechanisms of pathogenesis and facilitating advancements in diagnostics. However, when tackling this problem, existing machine learning and deep learning methods often face challenges such as data noise interference and inadequate integration of global and local information, leading to limited predictive performance.

To tackle these obstacles, we propose an innovative predictive framework called HGTMDA, which skillfully integrates random walk with restart-based association masking and a GCN-Transformer encoder for hypergraph learning. When processing the constructed similarity networks for each entity type, a random walk with restart-based association masking mechanism is introduced. By randomly masking partial association information, the model effectively mitigates issues related to data noise and insufficient information utilization. Simultaneously, the GCN-Transformer encoder is employed for hypergraph learning, enabling efficient integration of local and global information and enhancing the efficiency of information propagation and aggregation. The encoder gradually extracts and combines features at different scales through multiple stacked layers, improving the model’s expressive power. Furthermore, we utilize the DCE loss function to guide the model optimization process, taking into account both class imbalance and probability distribution differences. This approach allows the model to more effectively capture the inherent patterns within the data, significantly improving predictive performance. To comprehensively evaluate the model’s performance, we conducted five-fold cross-validation and feature ablation experiments. The results demonstrate that our proposed method achieves outstanding performance on multiple datasets, confirming its robustness and effectiveness. Moreover, we specifically analyzed the model’s predictions on lung cancer and colorectal cancer datasets. The results of these case studies reaffirm the remarkable advantages of our approach in uncovering the crucial roles of miRNAs in disease pathology.

Despite its strengths, HGTMDA has certain limitations. One notable issue is the lack of consensus on how to compute similarity scores between miRNAs and diseases, with various approaches leading to different results. These discrepancies in similarity scores can, to varying extents, impact model performance. Given the intricacy of this matter, our similarity analysis remains at a superficial level. In the future, we plan to further optimize the method, expand the data scale, and explore its potential applications in other diseases. Moreover, we intend to integrate HGTMDA with other omics data to develop multi-omics prediction models, aiming to obtain more comprehensive and accurate prediction results. We believe that HGTMDA provides new insights into miRNA–disease association research and will contribute to the advancement of precision medicine.

## Figures and Tables

**Table 1:** Comparison with other methods on HMDD v3.2.

Method	ACC	F1 Score	Recall	Precision	AUC	AUPRC
NIMCGCN	0.8131	0.8148	0.8220	0.8076	0.8945	0.8926
AGAEMD	0.8502	0.8507	0.8544	0.8481	0.9270	0.9286
MINIMDA	0.8481	0.8482	0.8529	0.8505	0.9304	0.9350
MAGCN	0.8483	0.8473	0.8425	0.8533	0.9245	0.9268
AMHMDA	0.8669	0.8653	0.8549	0.8763	0.9422	0.9411
HGTMDA	**0.8895**	**0.8920**	**0.8950**	**0.8890**	**0.9507**	**0.9492**

The best values are highlighted in bold.

**Table 2:** Comparative evaluation of alternative approaches.

Method	HGT-A	HGT-B	HGT-C	HGT-D	HGTMDA
AUC	0.9411	0.9398	0.9392	0.9482	**0.9507**
AUPR	0.9402	0.9384	0.9383	0.9467	**0.9492**

The best values are highlighted in bold.

**Table 3:** List of the top 20 miRNAs predicted to have the highest associations with lymphoma and lung cancer.

Cancer	Top 20 Prediction					
	Rank	miRNA	Evidence	Rank	miRNA	Evidence
Lung cancer	1	hsa-mir-155	dbDEMC	11	hsa-mir-218	dbDEMC
	2	hsa-mir-21	dbDEMC	12	hsa-mir-20b	dbDEMC
	3	hsa-mir-17	dbDEMC	13	hsa-mir-192	dbDEMC
	4	hsa-mir-126	dbDEMC	14	hsa-mir-34a	dbDEMC
	5	hsa-mir-20a	dbDEMC	15	hsa-mir-133a	dbDEMC
	6	hsa-mir-145	dbDEMC	16	hsa-mir-146a	dbDEMC
	7	hsa-mir-601	dbDEMC	17	hsa-mir-15a	dbDEMC
	8	hsa-mir-223	dbDEMC	18	hsa-mir-200b	dbDEMC
	9	hsa-mir-424	dbDEMC	19	hsa-mir-339	dbDEMC
	10	hsa-mir-106b	dbDEMC	20	hsa-mir-31	dbDEMC
Colorectal cancer	1	hsa-mir-21	dbDEMC	11	hsa-mir-10b	dbDEMC
	2	hsa-mir-146a	dbDEMC	12	hsa-mir-126	dbDEMC
	3	hsa-mir-34a	dbDEMC	13	hsa-mir-29a	dbDEMC
	4	hsa-mir-143	dbDEMC	14	hsa-mir-210	dbDEMC
	5	hsa-mir-145	dbDEMC	15	hsa-mir-100	dbDEMC
	6	hsa-let-7b	dbDEMC	16	hsa-mir-106a	dbDEMC
	7	hsa-mir-133a	dbDEMC	17	hsa-mir-451	dbDEMC
	8	hsa-mir-92b	dbDEMC	18	hsa-mir-196a	dbDEMC
	9	hsa-mir-17	dbDEMC	19	hsa-let-7a	dbDEMC
	10	hsa-mir-92a	dbDEMC	20	hsa-mir-20a	dbDEMC

## Data Availability

The miRNA–disease association data used in this study was obtained from the publicly available HMDD v3.2 database, through the website http://www.cuilab.cn/hmdd (accessed on 28 February 2024).

## Supplementary Materials

The following supporting information can be downloaded at: https://www.mdpi.com/article/10.3390/bioengineering11070680/s1, S1: Construction of various similarity networks for miRNAs and diseases, References [20,34,35,36,37] are cited in the supplementary materials.
