# Supplementary material for: HGTMDA: A Hypergraph Learning Approach with Improved GCN-Transformer for miRNA–Disease Association Prediction
Source: Bioengineering (Basel). 2024 Jul 4;11(7):680. doi: 10.3390/bioengineering11070680 (PMC11273495; doi:10.3390/bioengineering11070680)
Supplement: Supplementary file 1 [file bioengineering-11-00680-s001.zip › bioengineering-3026605-supplementary.pdf]

supplementary materials

S1: Construction of various similarity networks for miRNAs and diseases

In our study, we comprehensively evaluated the similarity between miRNAs and diseases from multiple perspectives, including miRNA functional similarity, sequence similarity, disease semantic similarity, and Gaussian interaction profile kernel similarity.

1.1 MiRNA Functional Similarity

Most current methods for calculating miRNA functional similarity rely on the MISIM database. To overcome the limitations of the MISIM database regarding miRNA functional similarity, we followed the approach of previous work [20]. This method cleverly combines the miRNA-gene interaction network and the weighted gene functional interaction network to compute miRNA functional similarity scores. The gene functional interaction network is derived from HumanNet [34], and the log-likelihood score (LLS) is used to quantify the association strength between genes. The functional similarity between m_i and m_j is as follows:

$$S_{mf}(m_i,m_j) = \frac{\sum_{h\in H_i} S(h,H_j)+\sum_{h\in H_j} S(h,H_i)}{|H_i|+|H_j|} \quad (1)$$

Here, H_i and H_j represent the datasets associated with m_i and m_j, respectively, while $|H|$ denotes the number of genes in the gene set. S_{mf} is the miRNA functional similarity matrix.

1.2 MiRNA Sequence Similarity

The sequence similarity of miRNA seed regions can be calculated by applying the Needleman-Wunsch algorithm [35]. This algorithm uses dynamic programming to find the optimal global alignment between two sequences, effectively quantifying the similarity between them. The miRNA sequence similarity is as follows:

$$S_{ms}(m_i,m_j) = \begin{cases} 1, & m_i = m_j \\ MS_N(m_i,m_j), & m_i \neq m_j \end{cases} \quad (2)$$

Here, $MS_N(m_i,m_j)$ is obtained by applying min-max normalization to the sequence similarity scores, and S_{ms} is the miRNA sequence similarity matrix.

1.3 Disease Semantic Similarity

According to previous research [36], Medical Subject Headings (MeSH) descriptors can be used to infer the semantic similarity between diseases. Using MeSH, we constructed a directed acyclic graph (DAG) to characterize the hierarchical relationships among diseases. Specifically, the DAG of disease d_f is represented as $DAG(d_f) = (d_f,T(d_f),E(d_f))$, where $T(d_f)$ denotes the set consisting of node d_f and its ancestor nodes, and $E(d_f)$ represents the set of edges between nodes in $DAG(d_f)$. Based on this, we define the semantic contribution measure of disease d_s to d_f as follows:

$$D_{d_f}(d_s) = \begin{cases} 1, & d_s = d_f \\ \max\{\Delta * D_{d_f}(d_s')|d_s' \in \text{children of } d_s\} & d_s \neq d_f \end{cases} \quad (3)$$

In the above formula, Δ represents the semantic contribution factor, which is usually set to 0.5 based on previous research. This setting implies that the semantic contribution of disease d_s to disease d_f decays exponentially as their distance in the DAG increases. Based on this definition, we further introduce the semantic value of disease d_f, denoted as $DS(d_f)$, which is the sum of the semantic contributions of all ancestor nodes of d_f. The mathematical expression is as follows:

$$DS(d_f) = \sum_{d_s \in T(d_f)} D_{d_f}(d_s) \quad (4)$$

According to the hypothesis of disease semantic similarity, the semantic similarity between diseases d_i and d_j is closely related to the semantic values of their common ancestor nodes in the DAG. In other words, if two diseases share more ancestor nodes with high semantic values, their semantic similarity score will be higher. Based on this principle, we define the semantic similarity between diseases d_i and d_j, with the mathematical expression as follows:

$$S_{ds}\left(d_i, d_j\right) = \frac{\sum_{d_t \in T(d_i) \cap T(d_j)} \left(D_{d_i}(d_f) + D_{d_j}(d_f)\right)}{DS(d_i) + DS(d_j)} \quad (5)$$

Here, S_{ds} is the disease semantic similarity matrix.

1.4 Gaussian interaction profile kernel Similarity for MiRNAs and Diseases

To further enrich the similarity information, we introduce the Gaussian Interaction Profile (GIP) to measure the similarity between miRNAs and diseases [37]. The basic assumption of GIP is that phenotypically similar diseases are more likely to be associated with functionally similar miRNAs. Based on this assumption, we define the GIP similarity between miRNAs m_i and m_j, with the mathematical expression as follows:

$$S_{mg}(m_i, m_j) = \exp\left(-\frac{\|\mathrm{IP}(m_i) - \mathrm{IP}(m_j)\|^2}{2\sigma_m^2}\right) \quad (6)$$

Here, $\mathrm{IP}\ (m_i)$ represents the miRNA-disease association matrix of the associations between m_i and each disease in m_j. σ_m is used to control the kernel bandwidth, with the mathematical expression as follows:

$$\sigma_m = \sqrt{\frac{\frac{1}{N_m}\sum_{i=1}^{N_m} \|\mathrm{IP}(m_i)\|^2}{2\gamma'_m}} \quad (7)$$

Here, γ'_m is measured by the average number of associations between all diseases and miRNAs, and following previous research, it is set to 1.0. The parameter N_m represents the number of miRNAs in the miRNA-disease association matrix. S_{mg} is the GIP similarity of miRNAs.

Similarly, the GIP similarity between diseases can be expressed mathematically as follows:

$$S_{dg}(d_i, d_j) = \exp\left(-\frac{\|\mathrm{IP}(d_i) - \mathrm{IP}(d_j)\|^2}{2\sigma_d^2}\right) \quad (8)$$

parameter σ_d is calculated as follows:

$$\sigma_d = \sqrt{\frac{\frac{1}{N_d}\sum_{i=1}^{N_d} \|\mathrm{IP}(d_i)\|^2}{2\gamma'_d}} \quad (9)$$

Here, S_{dg} represents the disease GIP similarity.

1.5 Target-based disease similarity

The target-based disease similarity is calculated using disease-gene interactions. with the mathematical expression as follows:

$$S_{dt}(d_i, d_j) = \frac{\sum_{h \in H_i} S(h, H_j) + \sum_{h \in H_j} S(h, H_i)}{|H_i| + |H_j|} \tag{10}$$

Here, H_i and H_j represent the datasets associated with d_i and d_j, respectively, while $|H|$ denotes the number of genes in the gene set. S_{dt} is the target-based disease similarity.
